# The STING/TBK1/IRF3/IFN type I pathway is defective in cystic fibrosis

**DOI:** 10.3389/fimmu.2023.1093212

**Published:** 2023-02-27

**Authors:** Luca Occhigrossi, Federica Rossin, Valeria Rachela Villella, Speranza Esposito, Carlo Abbate, Manuela D’Eletto, Maria Grazia Farrace, Antonella Tosco, Roberta Nardacci, Gian Maria Fimia, Valeria Raia, Mauro Piacentini

**Affiliations:** ^1^ Department of Epidemiology, Preclinical Research and Advanced Diagnostics, National Institute for Infectious Diseases IRCCS ‘L, Spallanzani’, Rome, Italy; ^2^ Department of Biology, University of Rome ‘Tor Vergata’, Rome, Italy; ^3^ European Institute for Research in Cystic Fibrosis, at National Institute for Infectious Diseases IRCCS ‘L, Spallanzani’, Rome, Italy; ^4^ Pediatric Unit, Department of Translational Medical Sciences, Regional Cystic Fibrosis Center, Federico II University Naples, Naples, Italy; ^5^ Department of Molecular Medicine, University of Rome “La Sapienza”, Rome, Italy; ^6^ Departmental Faculty of Medicine and Surgery, UniCamillus-Saint Camillus International University of Health and Medical Sciences, Rome, Italy

**Keywords:** cystic fibrosis, bacterial infections, innate immune response, STING pathway, type I interferon

## Abstract

Cystic fibrosis (CF) is a rare autosomal recessive disease caused by mutations in the cystic fibrosis transmembrane conductance regulator (CFTR) gene. The most common mutation is F508del-CFTR (ΔF) which leads the encoded ion channel towards misfolding and premature degradation. The disease is characterized by chronic bronchopulmonary obstruction, inflammation and airways colonization by bacteria, which are the major cause of morbidity and mortality. The STING pathway is the main signaling route activated in the presence of both self and pathogen DNA, leading to Type I Interferon (IFN I) production and the innate immune response. In this study, we show for the first time the relationship existing in CF between resistant and recurrent opportunistic infections by *Pseudomonas aeruginosa* and the innate immunity impairment. We demonstrate through *ex vivo* and *in vivo* experiments that the pathway is inadequately activated in ΔF condition and the use of direct STING agonists, as 2′,3′-cyclic GMP-AMP (2’, 3’ cGAMP), is able to restore the immune response against bacterial colonization. Indeed, upon treatment with the STING pathway agonists, we found a reduction of colony forming units (CFUs) consequent to IFN-β enhanced production in *Pseudomonas aeruginosa* infected bone marrow derived macrophages and lung tissues from mice affected by Cystic Fibrosis. Importantly, we also verified that the impairment detected in the primary PBMCs obtained from ΔF patients can be corrected by 2’, 3’ cGAMP. Our work indicates that the cGAS/STING pathway integrity is crucial in the Cystic Fibrosis response against pathogens and that the restoration of the pathway by 2’, 3’ cGAMP could be exploited as a possible new target for the symptomatic treatment of the disease.

## Introduction

Cystic Fibrosis (CF) is an autosomal recessive disease in which more than 2000 relevant mutations involving the gene coding for cystic fibrosis transmembrane conductance regulator (CFTR) were identified on chromosome 7 q31.2, and they are categorized in 6 classes according to their impact on the synthesis (class 1), processing (class Il), gating (class III), conductance (class IV), quantity (class V), and recycling (class VI) (1). CFTR is a 1480 amino-acid protein and belongs to ABC or ATP-binding cassette transporters and functions as chloride channel at apical membrane of epithelial cells. The channel has an important role in maintaining the epithelial surface hydrated and is found in liver, pancreas, intestine and lungs (2, 3). The most common mutation is the deletion of phenylalanine in position 508, F508del-CFTR (ΔF) (4, 5), which is a class II mutation and is responsible for 70% of CFTR loss-of- function mutations and is present in 90% of patients worldwide (1, 3, 6). The deletion causes protein misfolding that leads to a premature degradation and inability to reach the plasma membrane (7). Moreover, mutations of the CFTR lead to an altered activity of the channel causing an imbalance in the trans-epithelial fluid transport in various organs (8) and its most prevalent feature is the decreased muco-ciliary clearance with overproduction of thick and sticky mucus resulting in a chronic progressive lung disease. The latter is characterized by chronic inflammation, progressive obstructive pulmonary diseases, recurrent sinusitis and bronchitis, resistant and recurrent bacterial infections, mainly caused by *Pseudomonas aeruginosa (PA)* (1, 9, 10). The CF-associated bronchopulmonary disease is in fact the principal cause of morbidity and mortality. In the recent years the prognosis of CF has improved, owing to modern therapeutics and prevention of complications, in particular those regarding the respiratory system (11, 12). Recently, it has been demonstrated that the FDA approved drug Cysteamine, a known Tranglutaminase type 2 (TG2) inhibitor, has beneficial effects on patient affected by CF (7, 13). TG2 is a multifunctional protein involved in the pathogenesis of CF, in which the persistent and uncontrolled activity of the enzyme affects several processes as proteostasis, inflammation and autophagy, worsening the disease (14–19). In addition, it has been shown that TG2 knockout mice carrying the ΔF mutation were more resistant to bacterial infections and colonization by *PA* (16). Interestingly, a recent work showed that TG2 is involved in the host’s inflammatory response during bacterial infections, being able to modulate the type I Interferon (IFN I) production through the cGAS/STING pathway (20). The STING pathway is triggered by the recognition of exogenous nucleic acids by cGAS, a synthase that catalyses the formation of the cyclic dinucleotide 2’,3’ cyclic GMP-AMP (2’,3’cGAMP) from ATP and GTP (21–23). The newly formed 2’, 3’ cGAMP acts as second messenger binding STING and inducing TBK1/IRF3 signaling axis activation. TBK1 phosphorylates IRF3 at Serine 396 inducing its homodimerization and translocation in the nucleus, where it acts as transcription factor promoting the IFN-β expression (24). Following IFN-β production, JAK proteins bound to intracellular chains of IFN receptors are activated, auto phosphorylating themselves and STATs proteins. Phosphorylated STATs translocate in the nucleus and regulate IFN-stimulated genes (ISGs) expression, boosting the antimicrobial response (24–26). The lack of TG2 led to an upregulation of the STING pathway in the bone marrow-derived macrophages (BMDMs), explaining the increased antimicrobial activity observed in TG2 knockout mice (20). Recent years have seen a rapidly growing interest in the development of synthetic and natural STING-nucleotidic agonists. These cyclic dinucleotides (CDNs) act as immunomodulatory agent, capable of inducing an “antimicrobial state” boosting the INF-β production. Therefore, the CDNs may represent a novel drug against different pathogens infection (27). In this study we investigated whether a defective STING pathway could be responsible for the inadequate immune response to bacterial infection observed in CF patients.

## Material and methods

### Mice and treatments

FVB\129 mice wild type (CFTR^wt/wt^), heterozygous (CFTR^wt/F508del^) and homozygous (CFTR^F508del/F508del^) used for *ex vivo* experiment were littermates and they were obtained from Bob Scholte, Erasmus Medical Center Rotterdam, The Netherlands (CF-coordinated action program EU FP6 LSHM-CT-2005-018932). In order to prevent intestinal obstruction CF mice were fed with a special diet with high protein content (V1124-703Maus-Zucht, Charles River, Calco, Italy) and was replaced daily. Demineralized and acidified water was supplied ad libitum. Non-CF mice were fed with standard diet 4RF25, Mucedola, Milan, Italy) ad libitum. All mice were specific pathogen-free, maintained under a 12-h light/dark-cycle (7:00 to 19:00). Mice of all genotypes were age-matched at 7–9 weeks old at the beginning of infections. To perform *in vivo* experiment, CFTR^F508del/F508del^ (ΔF) mice were anesthetized with Ketamine 100 mg/Kg (Ketavet) plus medetomidine hydrochloride 1 mg/Kg (Domitor), followed by 2 hours intratracheal administration with 2′3′-cGAMP (20 μg), in 80 μL PBS. Then, pre-treated and non-pre-treated mice were infected with *P. aeruginosa* strain PAO-1. The infection was by intratracheal injection of 5 × 10^6^ cells/mice for 4 hours. At the end of the treatment, mice were sacrificed, and lungs were collected for analysis. All the procedures in mice were approved by the local Ethics Committee for Animal Welfare and were carried out in strict respect of European and National regulations (909/2017-PR).

### Mouse lung tissues histology

Lung tissue samples were obtained from untreated and from cGAMP-treated mice. Specimens from lungs tissues were fixed in 10% neutral-buffered formalin, and routinely processed to paraffin blocks. Sections of tissues (4 μm) were stained with hematoxylin and eosin (H&E).

### BMDMs and treatments

Bone marrow cells were isolated from FVB\129 mice wild type (CFTR^wt/wt^), heterozygous (CFTR^wt/F508del^) and homozygous (CFTR^F508del/F508del^) (7-9 weeks old), by collecting femur and tibia. Bone marrow cells were collected using 22GA syringe: Dulbecco’s modified Eagle’s medium (DMEM) was forced inside the bones and the medium containing the extracted cells was collected. Cells were cultured in DMEM supplemented with 10% fetal bovine serum, 100 μg/ml streptomycin and 100 units/ml penicillin, at 37°C and 5% CO2 in a humidified atmosphere, conditioned with L929 medium to stimulate macrophages differentiation. On day 7, differentiated BMDMs were stimulated with 250 μM cysteamine (Sigma Aldrich, M9768) or 20 μg 2’,3’ cGAMP (1 mg/ml) (*In vivo*gen) and then infected, in presence or absence of drugs, with *Pseudomonas aeruginosa* PAO-1. Cysteamine treatment was performed overnight; 2,3’ cGAMP transfection was performed for 2 hours using Lipofectamine 2000 (Invitrogen) according to the manufacturer’s instructions. BMDMs were infected with PAO-1 multiplicity of infection (MOI) 60 bacteria/macrophage to evaluate after 10 minutes and after 4h the level of internalization and clearance respectively. At time points, 10 min or 4 hours, cells were washed twice with sterile phosphate-buffered saline (PBS) and previously treated for 10 minutes with gentamicin (100 μg/ml, Sigma Aldrich, G1397) to remove any extracellular bacteria and lysed to evaluate the number of CFUs. To analyse proteins and RNA BMDMs were similarly infected and lysed after 4 hours. To selectively inhibit STING/IFN I axis, H-151 (*In vivo*gen) was used 1μg/ml overnight.

### PBMCs isolation and treatment

Peripheral blood mononuclear cells (PBMCs) were obtained from four patients with a confirmed diagnosis of CF, including sweat chloride over 60 mmol/liter, homozygous for F508del (mean age 13,75yrs, range 13,2-17,6) in stable clinical conditions in regular follow-up at Regional Cystic Fibrosis Center Pediatric Unit, Department of Translational Medical Sciences, Federico II University Naples, Italy. Healthy subjects were included as controls. An informed consent was obtained from each donor and the study was approved by the local The Ethics Committee. PBMCs were isolated using lympholite (Cederlane, UK) density gradient overlaid by heparin blood diluted 1:1 in PBS and centrifuged (20 min at 1000 g). After being washed three times, PBMCs were resuspended in complete RPMI 1640 supplemented with 25 mM HEPES, 10% (v/v) heat-inactivated FBS, 100 U/ml penicillin, 100 mg/ml streptomycin, and 1% 2 mM l-glutamine. PBMCs were stimulated with 20 μg 2’,3’ cGAMP for 1 hours using Lipofectamine 2000 (Invitrogen) and infected with PAO-1 (MOI 1:30) for 2 hours.

### Bacteria

*P. aeruginosa* strain PAO-1 was used for these studies. Bacteria were kept frozen at −80°C in 10% glycerol. Bacteria were thawed and grown in broth LB (Sodium Chloride (Sigma Aldrich), bacto tryptone (BD), yeast extract (BD) overnight. The concentration of bacteria was determined by spectrophotometric reading at 600 nm.

### CFU evaluation

Bone marrow cells from WT or ΔF mice were isolated and seeded in dish. After 7 days of differentiation, BMDMs obtained were infected with PAO-1 (MOI 1:60) for 10 minutes or 4 hours, to evaluate the level of internalization and clearance. Briefly, the cells- PAO-1 infected, were washed twice with sterile phosphate-buffered saline (PBS) and treated with gentamicin (100 μg/ml, Sigma Aldrich, G1397) for 10 min to remove any extracellular bacteria and extensively washed. The cells were then left for 10 min or 4h and then lysed to evaluate the number of CFUs. The lysis was performed with a solution 0.2% Triton x-100 (Biorad, Milan, Italy 1610407) and then, plated in agar petri dish and left overnight at 37°C. The number of CFUs after 10 minutes indicated the internalization of PAO-1 in BMDMs, while at 4 hours CFUs were analyzed to evaluate the clearance percentage. The effective intracellular PAO-1 clearance by BMDMs, was determined by calculating the percentage of living bacteria after 4 h of culture with respect to the total amount of internalized bacteria. ΔF mice were infected with PAO-1 or vehicle for 4 hours and then sacrificed. 1ml PBS was used for homogenization of isolated lung. Dilutions 1:10 and 1:100 were performed and 100 μl of appropriately serial diluted lung homogenates samples was plated in agar Petri dish for overnight incubation at 37°C. Living PAO-1 bacteria were enumerated after 4h of infection expressed as number of CFUs.

### Protein extraction and dosage

Cell extracts were obtained by collecting the medium from the flasks, washing the cells twice with PBS, detaching them with trypsin, and then blocking with the previously collected medium. The cells were subsequently centrifuged at 1400 rpm for 8 min and 28 the pellet was washed with PBS and centrifuged again, twice. The pellet was subsequently resuspended in Lysis buffer (20 mM Tris-HCl pH 7.4, 150 mM NaCl, 1% Triton X-100), containing the protease inhibitors, to lysate the cells. The cell lysate was kept on ice for 30 min and then sonicated for 6 seconds. The tumours explanted from mice, were homogenized in a buffer containing: 50 mM Tris-HCl pH 7.4, 50 mM NaCl, 1% Triton X-100, 10% glycerol, 320 mM sucrose and protease inhibitors. The tissue lysate was centrifugated at 13000 g and the supernatant recovered. Protein concentration was measured by the Bradford method using BIO-RAD PROTEIN ASSAY solution (Biorad); The required amount of protein was prepared by adding NuPAGE LDS 35 Sample Buffer 4X (Thermo Fisher Scientific) with β- Mercaptoethanol (2.86M); then the sample was denatured at 95° C for 10 min.

### Western blotting

The protein extracts were separated on SDS-polyacrylamide gel.Electrophoretic running was performed at constant 120 V with a running buffer containing 25 mM Tris-base, 192 mM Glycine, 0.1% SDS. Then, the proteins were transferred to a 0.2 μm thick nitrocellulose membrane (Protran) by electrophoresis at 300 mA for 1h 30 min in a buffer consisting of 25 mM Tris-Base, 192 mM glycine and 20% methanol. The non-specific membrane binding sites were blocked with lyophilized milk or bovine serum albumin (BSA; Sigma), dissolved at 5% in T-PBS (PBS + 0.05% Tween20), for 1 h at room temperature. The membranes were then incubated with primary antibodies (diluted in a 1% milk or BSA solution in T-PBS) overnight at 4°C or for 2 hours at room temperature. After incubation, the nitrocellulose membrane was washed 3 times for 10 min with T-PBS. Subsequently, it was incubated for 1 h with the secondary antibody, diluted 1: 5000 (Biorad) in the 1% milk or BSA solution, and then washed again three times with T-PBS. Finally, the proteins, bound by the antibody, were detected by incubating the membrane with a chemiluminescent ECL solution (Millipore) and using the Imager 600 machine (Amersham).

### Antibodies

Primary antibodies used: Anti-IRF3 (D83B9) Cat# 4302 (Cell Signaling); anti-phospho- IRF3 (Ser396) (D601M) Cat#29047 (Cell Signaling); anti-STAT1 (D1K9Y) Cat# 14994 (Cell Signaling); anti-phospho-STAT1 (Tyr701) (58D6) Cat#9167 (Cell Signaling): anti- STING (D2P2F) Cat# 13647 (Cell Singaling); anti-TBK1/NAK (D1B4) Cat#3504 (Cell Signaling); anti-phospho-TBK1/NAK (Ser172) (D52C2) Cat#5483 (Cell Signaling); Anti-Actin (Sigma).

### RNA extraction and dosage

To extract RNA from BMDMs, collected cells were lysed in Trizol reagent (Invitrogen, Carlsbad, CA) and total RNA was extracted using Direct-ZolTM RNA MiniPrep Plus according to the manufacturer’s instructions. The same procedure was followed for explanted lungs, 30 mg of tissue were used for RNA extraction.

### RT- PCR

2 μg of RNA was reverse transcribed by using SensiFASTTM cDNA Synthesis Kit (Bioline) following the manufactories instructions and used in quantitative RT–PCR (q-RT PCR) experiment. A no reverse transcriptase control (NRT) was used as a negative control to assess eventual amount of DNA contamination present in the RNA preparation.

### Real time PCR

The cDNA obtained from the RT-PCR was amplified by Real time PCR, using specific primers.

-Mouse Actin related primers: Forward (5’-GGCTGTATTCCCCTCCATCG-3’), Reverse (3’-CCAGTTGGTAACAATGCCATGT-5’)

-Mouse IFN-β related primers: Forward: (5′‐TCCGAGCAGAGATCTTCAGGAA‐3′), Reverse: (3′‐TGCAACCACCACTCATTCTGAG-5’)

-Human Actin related primers: Forward: (5’-AGCGGGAAATCGTGCGTG-3’), Reverse (5’-CAGGGTACATGGTGGTGCC-3’)

-Human IFN-β related primers: Forward (5’-TGGGAGGCTTGAATACTGCCTCAA-3’), Reverse (5’-TCTCATAGATGGTCAATGCGGCGT-3’)

SensiFASTTM SYBR Hi-ROX Kit (Bioline) was used following manufacturer’s instructions. Thermocycling consisted of an initial polymerase activation step at 98°C for 5 min, and amplification was performed with 35 cycles of 95°C for 15 s, 68°C for 10 s and 72°C for 20 s with data acquisition at this stage and the reaction finished by the built‐in melt curve. The relative amounts of mRNA were calculated by using the comparative Ct method.

### Cytokine detection

Supernatants from PAO-1-infected BMDM cultures and homogenates lung tissue from mice were collected and the IFN-β protein expression was evaluated by the mouse IFN-β ELISA kit (Thermo Fisher 424001) according to the manufacturer’s protocol.

### Statistical analysis

GraphPad was used for statistical analysis. ImageJ64 software was used for densitometric analysis. Statistical significance was determined using the Student’s t‐test or one‐way ANOVA test. P‐value smaller than 0.05 (P < 0.05) was considered to be significant.

## Results

### The STING pathway is impaired in ΔF BMDMs

**Figure 1:**
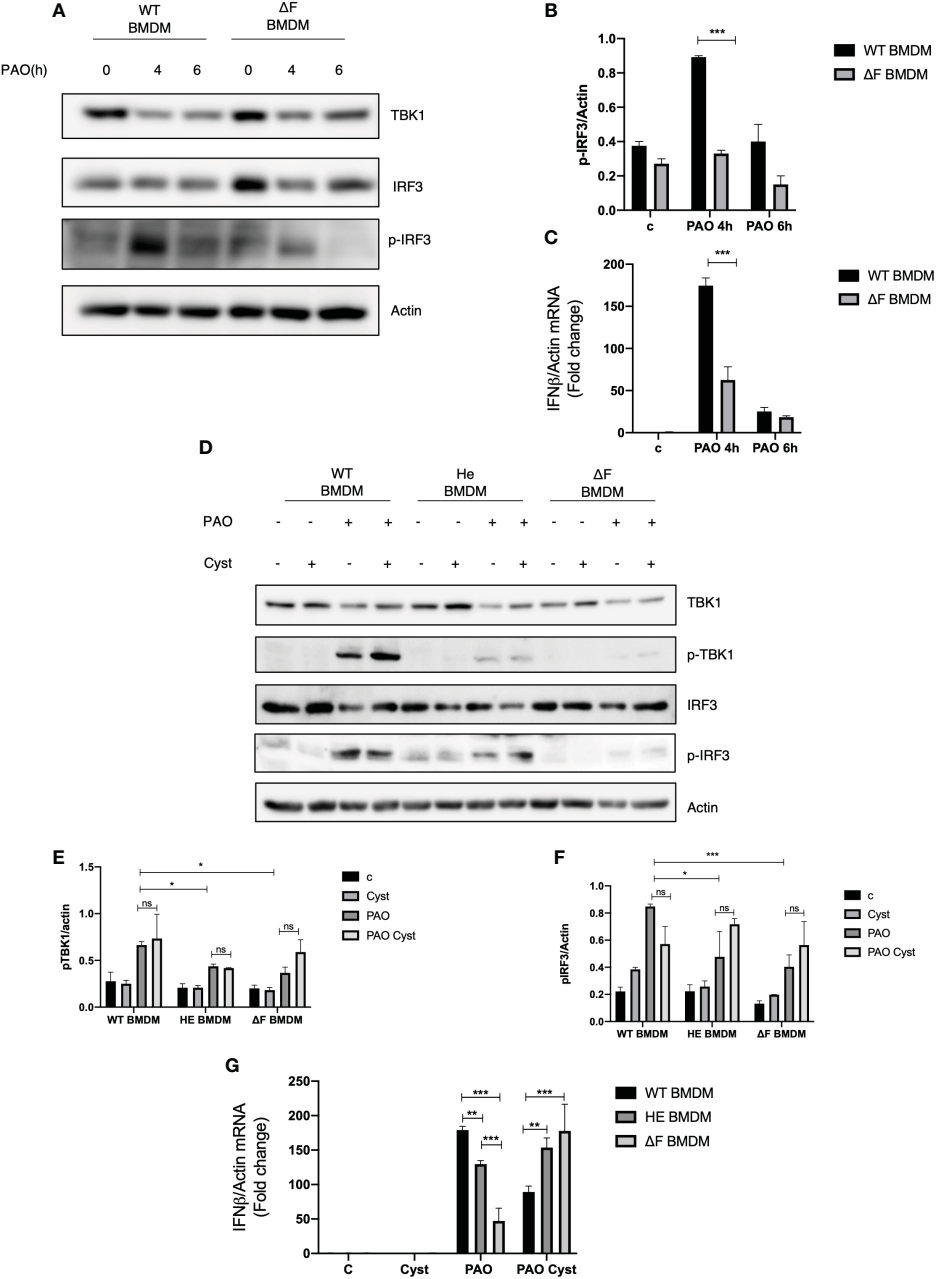
cGAS/STING pathway is downregulated in ΔF BMDMs **(A)** Western blot analysis of TBK1, IRF3 and p-IRF3 expression levels in WT and ΔF BMDMs after 4h and 6h infection with *Pseudomonas aeruginosa* strain (PAO-1). Actin was used as loading control. **(B)** Densitometric analysis showing p-IRF3 and actin expression levels in BMDMs following PAO-1 infection (n=3; means ± SEM; ***p<0.001). **(C)** IFN-β mRNA levels, quantified by qPCR, in BMDMs obtained from WT and ΔF mice after 4h and 6h infection with PAO-1, normalized to the mRNA levels of actin. (n=3; means ± SEM; ***p<0.001). **(D)** Western blot analysis of TBK1 and IRF3, and their phosphorylated forms, with their respective densitometric analysis **(E, F)**, in PAO-1 infected WT, HE and ΔF BMDMs following overnight cysteamine treatment. Actin was used to normalize protein loading levels. (n=3; means ± SEM; *p<0.05; ***p<0.001). **(G)** IFN-β mRNA expression levels, quantified by qPCR, in BMDMs treated with cysteamine, and infected with PAO-1 for 4h. Actin mRNA levels were used to normalize IFN-β mRNA levels. (n=3; means ± SEM; **p<0.01; ***p<0.001).

Recently we demonstrated that macrophages isolated from TG2 knockout mice displayed an upregulation of the cGAS/STING/TBK1 pathway, the main innate immunity routes involved in the early defence against bacterial infections (20). Considering that TG2 activity is upregulated in CF and that the enzyme negatively affects the STING signaling, we questioned whether CF mice could display a derangement in immune response contributing to bacteria colonization. To this aim, we infected primary BMDMs from WT and ΔF mice with *Pseudomonas Aeruginosa* PAO-1 strain, evaluating the effect of the infection after 4 and 6 hours. Firstly, we evaluated TBK1 and IRF3 in WT and ΔF BMDMs, and we did not find alterations in the protein levels among these cells (**Figure 1A**). Moreover, **Figure 1A** shows that 4 hours of infection is the optimal time to induce massive IRF3 phosphorylation at Serine 396, which is used as a marker of the STING pathway activation (28). Interestingly, we found that ΔF BMDMs showed a significant decreased in the p-IRF3 levels with respect to WT BMDMs, suggesting an impairment of the cGAS/STING axis in the CF condition (**Figures 1A, B**). In order to confirm the deficient activation of the pathway in ΔF, we analyzed the mRNA expression levels of the IFN-β produced by the BMDMs in response to PAO-1 infection (**Figure 1C**). The Real time PCR indicated that after 4 hours of infection the IFN-β expression was significantly reduced in ΔF BMDMs compared to WT. Hence, these results suggest that the ΔF mutation leads to an impairment in the cGAS/STING pathway activation, possibly due to upregulation of the TG2 activity reported in CF disease (17, 29). Cysteamine is a known TG2 inhibitor capable of restoring a functional CFTR in ΔF BMDMs and it is endowed with bactericidal activity against PAO-1 (7). Thus, we questioned if cysteamine could also engage the cGAS/STING pathway stimulation. To this aim, WT, heterozygous (HE) and homozygous ΔF BMDMs were treated with cysteamine followed by 4 hours of exposure to PAO-1 infection. Interestingly, our data show that p-TBK1 and p-IRF3 levels were already decreased in heterozygous (HE) and more evidently in the ΔF genotypes, showing that the mutation in only one allele leads to the STING pathway downregulation (**Figures 1D–F**). Moreover, **Figures 1D-F** display that cysteamine treatment did not promote changes in the protein phosphorylation levels of TBK1 and IRF3, indicating that the drug is not able to affect the STING pathway. However, being cysteamine able to evoke protection against invading pathogens, we verified if the drug could modulate the IFN-β expression. In **Figure 1G** we showed an increase in INF-β production in WT compared to ΔF BMDMs, confirming our previous results. In addition, we observed that HE BMDMs displayed an intermediate IFN-β expression with the respect to WT and ΔF cells indicating that the functional CFTR protein plays a key unknown role in the IFN response. Interestingly, after cysteamine administration ΔF BMDMs showed an increase of INF-β expression, while the cytokine production was decreased in WT BMDMs. Taken together, these results indicate that cysteamine is able to modulate the IFN-β expression in a STING pathway independent way.

### 2’, 3’ cGAMP restores the STING pathway *ex vivo*

**Figure 2:**
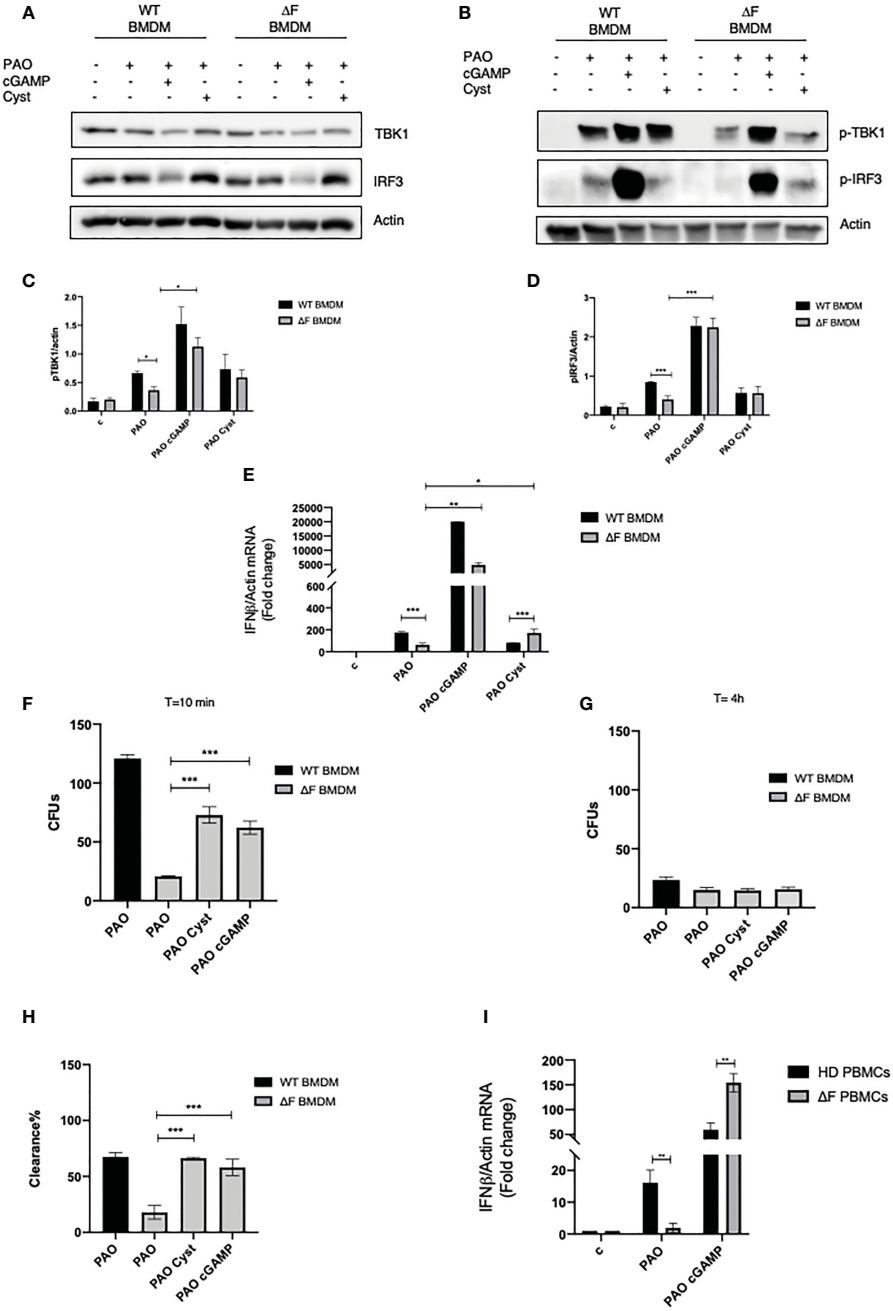
2’,3’ cGAMP restores the STING axis in ΔF *ex vivo* models **(A)** Western blot analysis of TBK1 and IRF3, and their phosphorylated forms **(B)**, with their respective densitometric analysis **(C, D)**, in PAO-1 infected WT and ΔF BMDMs, following overnight cysteamine treatment and 2h of 2’,3’ cGAMP stimulation. Actin was used to normalize protein loading levels. (n=3; means ± SEM; *p<0.05; ***p<0.001). **(E)** IFN-β mRNA levels, quantified by qPCR, in WT and ΔF BMDMs treated with cysteamine and 2’,3’cGAMP. Actin mRNA levels were used to normalize IFN-β mRNA levels (n=3; means ± SEM; ***p<0.001). **(F)** PAO-1 internalization at T=10 expressed as number of CFUs. **(G)** Living PAO-1 after 4 hours expressed as number of CFUs. **(H)** Percentage of PAO-1 clearance expressed as living bacteria after 4 hours of culture with respect to internalized bacteria. (n=3; means ± SEM; ***p<0.001). **(I)** IFN-β mRNA levels, quantified by qPCR, in Human PBMCs from ΔF (n=4) and healthy donors (HD) (n=4). Cells were stimulated with 2’,3’ cGAMP for 1h and then infected with PAO-1 for 2h. The mRNA levels of IFN-β were normalized to the mRNA levels of actin. (**p<0.01).

Prompted by the above reported data showing the presence of a defective cGAS/STING/TBK1/IFN-β pathway in ΔF BMDMs we verified whether the direct stimulation of the cGAS/STING by the 2’,3’cGAMP agonist was able to rescue the IFN-β production. To this aim, WT and ΔF cells were treated with 2’, 3’ cGAMP followed by PAO-1 infection. As shown in **Figures 2A-D** 2’, 3’ cGAMP was able to increase TBK1 and IRF3 phosphorylation in ΔF BMDMs, thus restoring the STING pathway activation. Subsequently, we analyzed the IFN-β mRNA expression in PAO-1 infected BMDMs, upon the direct STING pathway stimulation. We compared IFN-β expression induced by the STING agonists to the one evoked by cysteamine treatment. **Figure 2E** shows that the treatment with 2’, 3’ cGAMP is able to markedly enhance the IFN-β levels compared to those induced without STING agonists, both in WT and ΔF BMDMs, indicating a functional re-establishment of the cGAS/STING axis in CF cells. Interestingly, 2’, 3’ cGAMP boosted significantly IFN-β expression in ΔF up to fifty folds more than cysteamine treatment. It has been shown that CF mice display a defective bacterial internalization and a consequent impairment in the clearance process (30). Being cysteamine able to enhance bacterial clearance (31), we analyzed whether the 2’,3’ cGAMP could mediate the same effect. To this aim, we evaluated PAO-1 internalization at early time, followed by clearance analysis, expressed as number of CFUs. The results in **Figures 2F-H** show that 2’, 3’ cGAMP treatment is effective to improve internalization and clearance of PAO-1 by BMDMs of CF mice, indicating that the stimulation of STING pathway increases the ability of ΔF macrophages to reduce bacterial proliferation. However, to assess if the STING/IFN-β axis is crucial for bacterial clearance, we treated WT and ΔF BMDM with the selective STING inhibitor H-151. As shown in **Supplementary Figures 1A-C**, H-151 was able to shut down both IRF3 phosphorylation and IFN-β levels upon 2’, 3’ cGAMP stimulation. Interestingly, the inhibition of the STING pathway turned out in a marked reduction of PAO-1 internalization and clearance in ΔF BMDM, confirming that the stimulation of the STING/IFN-β axis is necessary in CF condition to solve the infection (**Supplementary Figures 1D–F**). Afterward, we verified whether the defective STING axis could be a common feature of the human peripheral blood mononuclear cells (PBMCs) obtained from CF patients. In fact, they consist of lymphocytes and monocytes that are highly responsive upon infection (32, 33). Thus, PBMCs were isolated from CF patients carrying ΔF mutation, and healthy volunteers, and then infected with PAO-1 for 2h. 2’,3’ cGAMP treatment was performed for 1h in order to stimulates the STING pathway. **Figure 2I** shows that ΔF PBMCs display a reduced IFN-β response following PAO-1 infection, compared to WT. As expected, the 2’,3’ cGAMP stimulation led to enhanced IFN-β expression in ΔF cells. This result indicates that the alteration of the STING pathway occurs not only in murine BMDMs, but also in human PBMCs, and that the stimulation with 2’3’ cGAMP is able to increase the IFN-β expression in CF patients derived cells.

### The STING agonist stimulates autophagy in ΔF BMDMs

**Figure 3:**
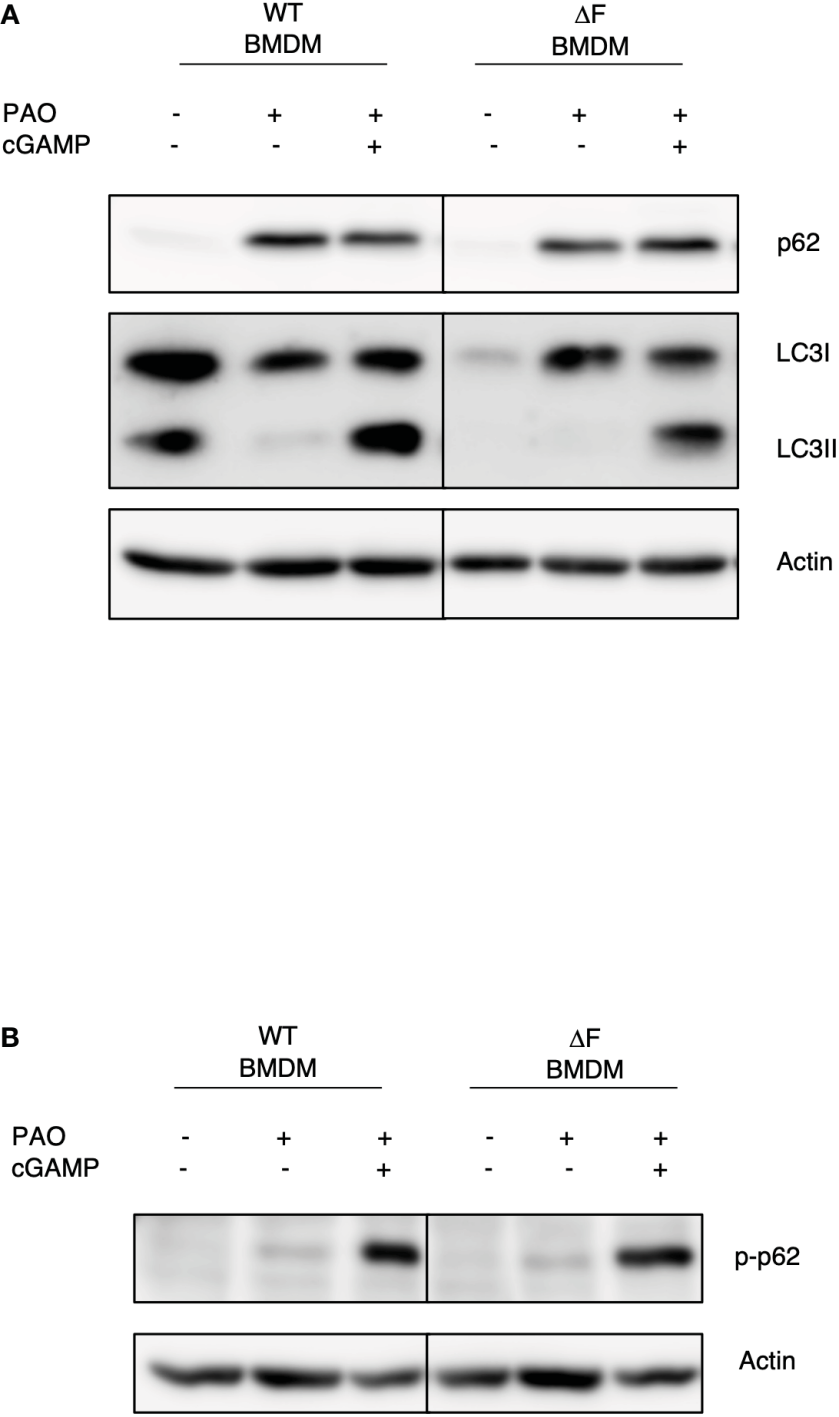
The re-establishment of the STING axis promotes autophagy in ΔF BMDMs **(A)** Western blot analysis of P62 and LC3I/II in PAO-1 infected WT and ΔF BMDMs, following 2h of 2’,3’ cGAMP stimulation. **(B)** Western blot analysis of p-P62 in PAO-1 infected BMDMs.

It has been reported that the impaired bacterial handling by macrophages is a key feature of CF airways, caused by defective autophagy (30). Thus, we wanted to understand whether restoring the cGAS/STING axis activity, due to the administration of 2’,3’ cGAMP, could also lead to a re-establishment of the autophagy in ΔF BMDMs. To this aim, BMDMs from WT and ΔF mice were infected with PAO-1 and treated with 2’,3’ cGAMP. **Figure 3A** shows that the PAO-1 infection stimulates a robust p62 expression, indicating the involvement of xenophagy during the infection. Interestingly, the STING agonist was able to stimulate p62 phosphorylation at serine 403, which is a key marker of autophagy induction, in both WT and ΔF BMDMs (**Figure 3B**). In addition, we found that 2’,3’ cGAMP promoted LC3I/LC3II conversion (**Figure 3A**), not only in WT, but also in CF cells that displayed a defective autophagy process. In fact, WT cells showed high LC3II protein levels in basal conditions, which undergo to a reduction upon infection, indicating a degradation inside the autophagolysosomes. Instead, the LC3II protein expression was undetectable in CF BMDMs both in basal condition and upon infection, confirming the defect in the process. Interestingly, 2,’3’ cGAMP was able to stimulate the LC3II formation, thus promoting autophagy induction. These results suggest that the rescued function of ΔF macrophages upon 2’,3’ cGAMP treatment, could be mediate by xenophagy, stimulated by the STING pathway.

### *In vivo* administration of 2’, 3’ cGAMP restores the STING pathway in infected ΔF mice

**Figure 4:**
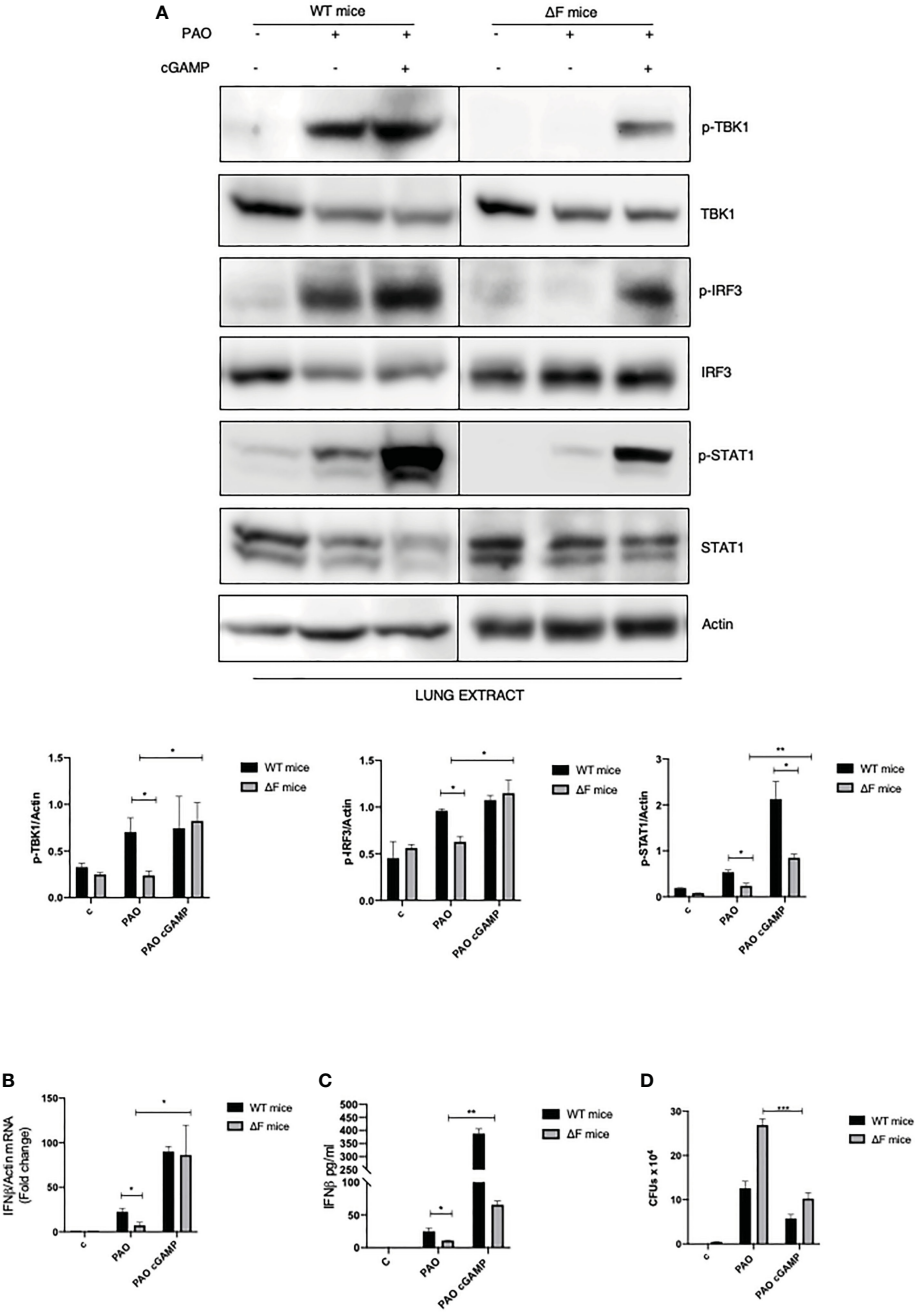
2’,3’ cGAMP restores the STING axis increasing bacterial removal in ΔF mice **(A)** Western blot analysis of IRF3, TBK1, STAT1 and their phosphorylated forms, with their respective densitometric analysis, in the lungs of WT and ΔF mice subjected to 2’,3’ cGAMP pre-treatment and to 4h PAO-1 infection. Actin was used as protein loading control. (n=4; means ± SEM; *p<0.05; **p<0.01). **(B)** IFN-β mRNA levels, quantified by qPCR, in lungs from PAO-1 infected in WT and ΔF mice following 2’,3’ cGAMP pre-treatment for 2h and then PAO-1 infected for 4h. The mRNA levels of IFN- β were normalized to the mRNA levels of actin. (n=4; means ± SEM; *p<0.05). **(C)** IFN-β protein expression quantified by ELISA, in lungs from PAO-1 infected in WT and ΔF mice following 2’,3’ cGAMP pre-treatment for 2h and then PAO-1 infected for 4h. (n=4; means ± SEM; *p<0.05; **p<0.01). **(D)** Enumeration of living PAO-1 bacteria in lung after 4h of infection expressed as number of CFUs (n=4; means ± SEM; ***p<0.001).

Following the *ex vivo* results obtained on BMDMs, we decided to focus on the *in vivo* system. Hence, we verified whether the defective STING pathway found in CF mice could be restored following the use of agonists correlating this to a concomitant reduction in bacterial proliferation. WT and ΔF mice were exposed for 2 hours to 2’, 3’ cGAMP pre-treatment and then intratracheally infected with PAO-1 for 4 hours. Interestingly, histological sections of lungs, from cGAMP-treated mice, showed normal respiratory epithelium, comparable to the alveolar epithelia from control mice, indicating that the 2’,3’ cGAMP treatment did not result to be harmful for the mice lung tissue (**Supplementary Figure 2**). As reported in **Figure 4A** p-TBK1 and p-IRF3 proteins were highly expressed in the lungs of infected mice subjected to 2’, 3’ cGAMP treatment compared to mice that did not receive the STING agonists. We also analyzed the downstream STAT1 phosphorylation which was enhanced in 2’, 3’ cGAMP treated ΔF mice as well as the IFN-β mRNA levels. **Figures 4B, C** clearly shows that the STING axis stimulation with 2’, 3’ cGAMP led to a significant increase in INF-β levels in infected ΔF mice. Furthermore, in order to verify whether the enhanced IFN-β response was strictly correlated with an amelioration in terms of defense against opportunistic infections, we evaluated the number of living PAO-1 bacteria in lungs from ΔF mice subjected to 2’, 3’ cGAMP administration (**Figure 4D**). In accordance with the above reported data obtained from *ex vivo* experiments, upon 2’, 3’ cGAMP treatment, the number of CFUs were reduced suggesting that the drug was effective to limit the PAO-1 infection in CF mice airways. Therefore, these data indicate that the direct STING stimulation, *in vivo*, effectively re-establish the activation of the STING axis, which is compromised in CF, counteracting bacterial proliferation.

## Discussion

Cystic fibrosis is a rare genetic disease caused by mutations on CFTR gene which encodes for a protein that functions as chloride ion channel, necessary to maintain correct trans- epithelial fluid transport. It is an autosomal recessive systemic disorder, having as main feature chronic respiratory impairment (1, 2, 34). The most common mutation is F508del-CFTR (ΔF) which causes misfolding of the protein and its degradation, with consequent inability to reach the plasma membrane (1, 3). According to literature, TG2 is overactivated by the high levels of misfolded proteins, ROS, increased Ca2+ flux and inflammation present in the epithelial cells derived from CF patients (15, 16, 18). TG2 is a multifunctional member of the transglutaminase family of proteins, implicated in different processes including the We showed that the In fact, CFTR stability is directly influenced by TG2, promoting the degradation of the channel by HSF1/HSP70 axis activation (16). Moreover, the persistent and uncontrolled activity of the enzyme is also related to high ROS production favoring the proinflammatory and prooxidative environment characterizing CF (15). CF airways are characterized by chronic inflammation and recurrent bacterial infections, mainly caused by *Pseudomonas aeruginosa*, the major opportunistic pathogen in the lung. TG2 knockout mice carrying the ΔF mutation were more resistant to bacterial infections and colonization by *Pseudomonas aeruginosa* PAO-1 strain (16). Considering that TG2 activity is upregulated in CF and that the enzyme negatively affects the STING signaling (20), we questioned whether CF mice could display a derangement in immune response contributing to bacteria colonization. *Ex vivo* experiments carrying out on BMDMs isolated from ΔF mice demonstrated, for the first time, that the mutation is linked to a decreased IRF3 phosphorylation at Ser 396 resulting in a defective IFN-β production in response to PAO-1 infection, meaning an inadequate activation of the pathway. The severity of this defect is also evidenced by the fact that already the mutation in one allele induces the STING pathway derangements. In fact, the phosphorylation levels of TBK1 and IRF3 progressively decreased from WT to heterozygous up to homozygous ΔF BMDMs. Moreover, upon stimulation, the heterozygous displayed an intermediate IFN-β production with respect to WT and ΔF BMDMs, suggesting that the fifty percent of the mutated CFTR is inadequate to trigger a physiological response, but it is sufficient to avoid the insurgence of the pathology and its related complications. Future studies must investigate how mutation of the CFTR is mechanistically linked to the STING pathway impairment. We also investigated the effects of cysteamine, which acting as proteostasis regulator, restores functional CFTR at plasma membrane and induces a reduction of CFUs in PAO-1 infected ΔF BMDMs (7). Interestingly, the cysteamine beneficial effects showed to be independent from the STING pathway, since treatment on BMDMs failed to induce changes in TBK1 and IRF3 levels. However, the IFN-β levels were enhanced in the cysteamine treated ΔF BMDMs suggesting that the reduction of CFUs could be possibly linked to IFN-β and the downstream effectors of the cytokine which are involved in antimicrobial activity. On the contrary, IFN-β production in the cysteamine treated WT BMDMs was interestingly reduced, indicating that the drug acts differentially based on genotype. In fact, cysteamine is a small molecule that influences cellular redox homeostasis and oxidative state, which regulates pathways involved in survival and gene expression (35, 36). Therefore, the opposite effect found could be linked to the inflammatory state displayed by CF cells, which in turn leads to a modification of redox state. In CF condition, the extensive ROS production can affect proteostasis, modifying several protein functions. In this scenario, cysteamine could interact with an altered protein network, interfering with the IFN production. Conversely, in normal condition, the inflammatory environment is reduced as well as the ROS production, resulting in a different protein homeostasis compared to CF. Cysteamine can affect in different ways the transcription of the cytokine resulting in its decreased expression. Interestingly, To this aim, we showed that the 2’,3’cGAMP was able to rescue the TBK1 as well as IRF3 phosphorylation in the defective ΔF BMDMs, and this was correlated with a marked increase of IFN-β expression. Furthermore, we defined that the ΔF BMDMs treated with 2’, 3’ cGAMP and infected with PAO-1 display both an enhanced bacterial internalization and clearance which is paralleled by a It is important to note that 2’, 3’ cGAMP was able to restore proper autophagy in CF cells. It is important to note that these, ex vivo, findings were confirmed in vivo in explanted lungs from PAO-1 infected ΔF mice. These evidence indicate that cGAMP displays double beneficial effects on CF by inducing autophagy and stimulating the innate immunity (37). Importantly, In fact, we successfully validated the possibility of rescuing the defective innate immunity STING pathway in PBMCs isolated from patients carrying the ΔF mutation. In conclusion, our data indicate for the first time the ΔF mutation is associated to a defective/silent STING pathway, one of key innate immunity routes against pathogens. However, Our study also demonstrates that the cGAS/STING pathway in ΔF mice can be restored by treatment with agonists and that its activation re-established the defences host response against opportunistic infections. Interestingly, 2’, 3’ cGAMP “per se” is not suitable for prolonged treatment in patients, due to its hydrolysis by the ENPP1 (38). In fact, it has a half-life in the range of hours, making it more efficient just during acute infection. Nevertheless, several analogs of 2’, 3’ cGAMP have been developed, displaying an increased biostability and they are currently under evaluation for clinical trials (39). Among these ADUS100 has reached the phase II without negative indications about its toxicity in vivo. Therefore, the restoration of the pathway in ΔF could be exploited as a possible target in the palliative treatment of the disease, since its correct functioning is fundamental in counteracting bacterial superinfections, especially of Pseudomonas Aeruginosa, which compromise cystic fibrosis patients’ condition and their life expectancy.

## Data availability statement

The raw data supporting the conclusions of this article will be made available by the authors, without undue reservation.

## Ethics statement

The studies involving human participants were reviewed and approved by Italian Ministry of Health. Written informed consent to participate in this study was provided by the participants’ legal guardian/next of kin. The animal study was reviewed and approved by Italian Ministry of Health.

## Author contributions

LO designed and performed most of the experiments. FR, MD and MF helped with the experiments. VV and SE performed the PAO1 infections on BMDMs and PBMCs. AT selected and enrolled CF patients and Healthy controls. CA performed PAO1 infection on mice. RN performed the analysis of lungs of histological sections. MP and LO wrote the paper. VR, GF and MP conceived the project. All authors contributed to the article and approved the submitted version.
