# Supplementary material for: The STING/TBK1/IRF3/IFN type I pathway is defective in cystic fibrosis
Source: Front Immunol. 2023 Feb 27;14:1093212. doi: 10.3389/fimmu.2023.1093212 (PMC10008931; doi:10.3389/fimmu.2023.1093212)
Supplement: Supplementary file 1 [file DataSheet_1.docx]

**
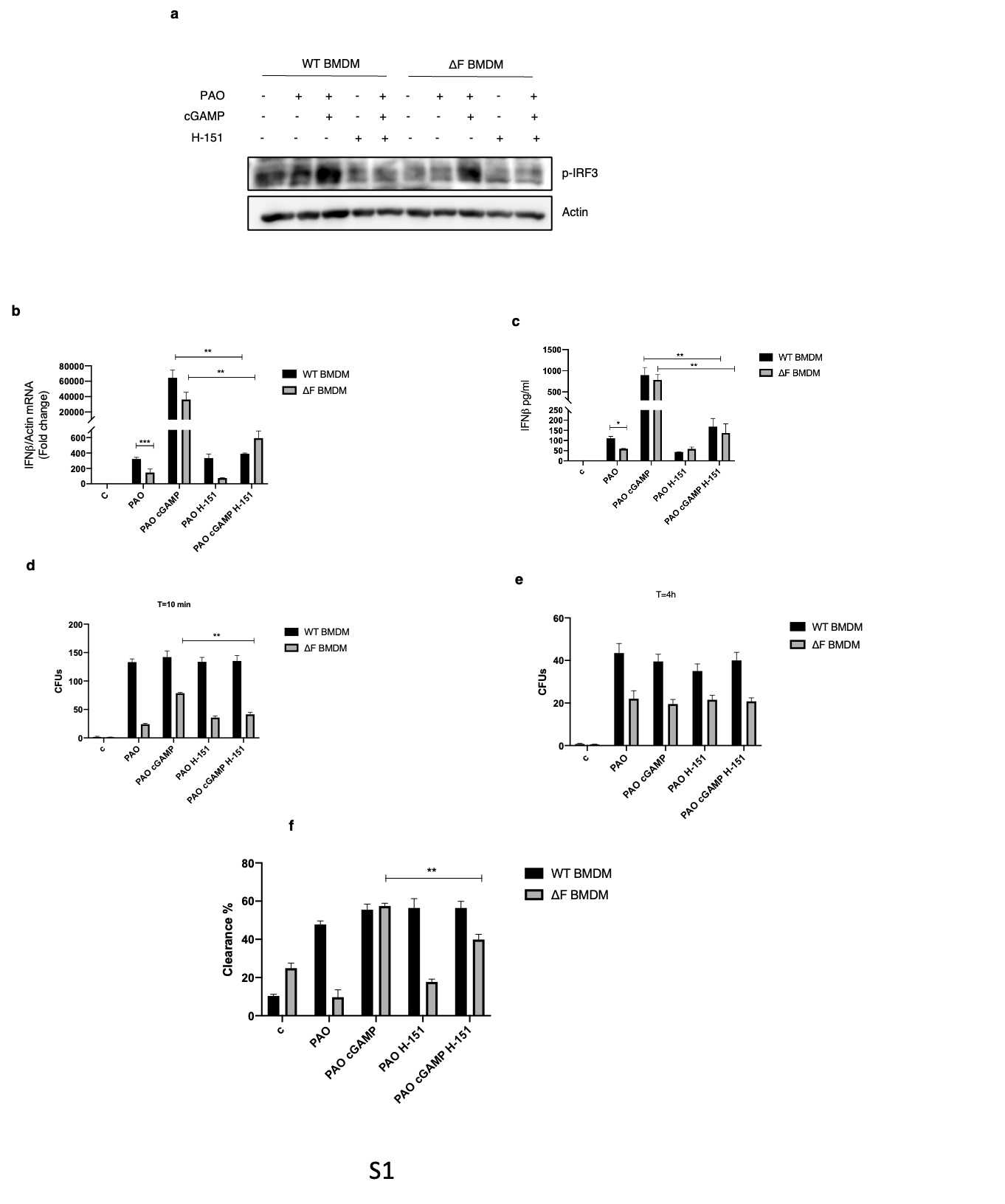
**

**Supplementary Figure 1. The inhibition of STING/IFN I axis impairs the bacterial clearance in ΔF BMDMs**

(A) Western blot analysis of p-IRF3 in PAO-1 infected WT and ΔF BMDMs, following H-151 overnight treatment and 2h of 2’,3’ cGAMP stimulation. Actin were used as loading control. (B) IFN-β mRNA levels, quantified by qPCR, in BMDMs obtained from WT and ΔF mice after infection with PAO-1, following H-151 overnight treatment and 2h of 2’,3’ cGAMP stimulation, normalized to the mRNA levels of actin. (**p<0.01). **(**C**)** IFN-β protein expression, quantified by ELISA, in the supernatant of WT and ΔF BMDMs after infection with PAO-1, following H-151 overnight treatment and 2h of 2’,3’ cGAMP stimulation. (D) PAO-1 internalization at T=10 expressed as number of CFUs. (E) Living PAO-1 after 4 hours expressed as number of CFUs. (F) Percentage of PAO-1 clearance expressed as living bacteria after 4 hours of culture with respect to internalized bacteria.


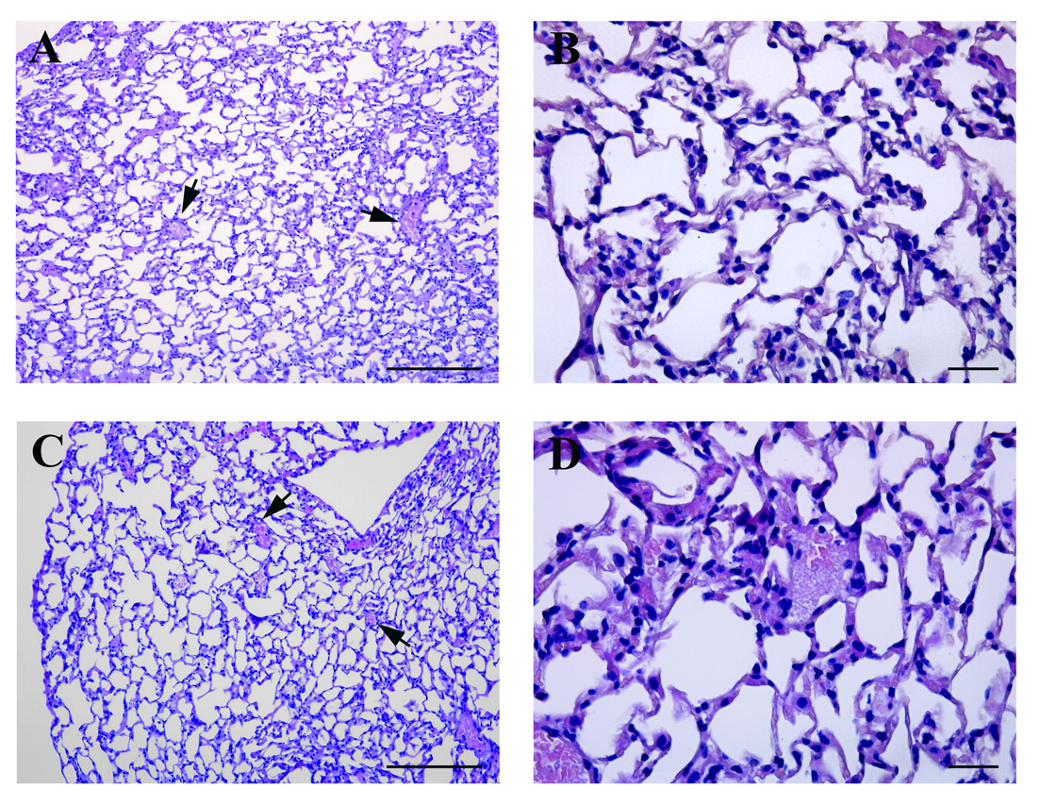


**Supplementary Figure 2. 2’,3’ cGAMP treatment *in vivo* does not harm the lung tissue**

(A and B) Hematoxylin and eosin staining of lung tissue from control mice shows normal respiratory epithelium. Capillaries containing red blood cells within their lumen, next to an endothelial cells, are visible (A, arrows). (C and D) Lung of histological sections from cGAMP-treated mice showing normal respiratory epithelium. Capillaries are visible (C, arrows). Higher magnification of cGAMP-treated lung tissue shows a well preserved alveolar epithelia. Scale bars: A,C 100 µm; B,D 14 µm.
